# Digital Rehabilitation Interventions in Sub-Saharan Africa: Protocol for a Scoping Review

**DOI:** 10.2196/48952

**Published:** 2023-11-23

**Authors:** Michael Oduor, Katariina Korniloff, Juliette Gasana, David K Tumusiime, Eeva Aartolahti

**Affiliations:** 1 Institute of Rehabilitation, Jamk University of Applied Sciences Jyväskylä Finland; 2 School of Health Sciences, University of Rwanda Kigali Rwanda

**Keywords:** digitalization, rehabilitation, scoping review, sub-Saharan Africa, telerehabilitation

## Abstract

**Background:**

Estimations show that at least one in every 3 people in the world needs rehabilitation at some point in the course of their illness or injury. Access to rehabilitation services is an essential part of the continuum of care and is integral to achieving universal health coverage. However, most of the world’s population living in low- and middle-income countries, especially in the sub-Saharan African region, does not have access to adequate rehabilitation services. Wider adoption of digital solutions offers opportunities to support and enhance access to rehabilitation services in sub-Saharan Africa. A region where there is a greater burden and need for these services. There is also little published research about digital rehabilitation in sub-Saharan Africa, as it is an underexamined topic in the region.

**Objective:**

This scoping review aims to provide a comprehensive picture of the current evidence of digital interventions in rehabilitation implemented in any health, social, educational, or community setting in the sub-Saharan Africa region.

**Methods:**

We will conduct a scoping review using Arksey and O’Malley’s methodological framework and follow the Joanna Briggs Institute methodology for scoping reviews. We will develop search strategies for a selected number of web-based databases, search for peer-reviewed scientific publications until September 2023, and screen the reference lists of relevant articles. We will include research articles if they describe or report the use of digital interventions in the rehabilitation of patients with any health problem or disability in sub-Saharan Africa. For selected articles, we will extract data using a customized data extraction form and use thematic analysis to compare the findings across studies.

**Results:**

The preliminary database search in MEDLINE (EBSCO) was completed in May 2023. The research team will conduct a search of relevant articles in the autumn. The results will be synthesized and reported under the key conceptual categories of this review, and we expect the final scoping review to be ready for submission in early 2024.

**Conclusions:**

We expect to find gaps in the research and a lack of detailed information about digital rehabilitation interventions in sub-Saharan Africa, as well as potential areas for further study. We will identify opportunities to inform the development of digital rehabilitation interventions.

**International Registered Report Identifier (IRRID):**

PRR1-10.2196/48952

## Introduction

### Background

Anyone with a health condition who experiences difficulties in any area of their life might need rehabilitation. According to the World Health Organization (WHO) [1], rehabilitation is defined as “a set of interventions designed to optimize functioning and reduce disability in individuals with health conditions in interaction with their environment.” By aiming to optimize functioning, rehabilitation can help an individual be as independent as possible in everyday activities and support individuals to participate in education, work, recreation, and other meaningful life roles [2-4].

It is estimated that at least one in every 3 people in the world needs rehabilitation at some point in the course of their illness or injury [4]. According to the Global Burden of Disease study [4], the need for rehabilitation has increased by 63% since 1990, rising from 1.48 billion to 2.41 billion people [4]. Thus, the demand for rehabilitation services already exceeds availability, leaving a large unmet need, especially in low- and middle-income countries (LMICs) [5]. This provides strong evidence to prioritize rehabilitation and address the functioning needs of the population.

Rehabilitation has been effective in improving functioning outcomes in different health conditions in low-income and lower-middle-income countries, but it has not been prioritized in some of these countries and is underresourced [4]. Strengthening and reforming systems for health and collaborative practice requires a reconsideration of the role of the “patient.” Patients are moving from being passive recipients to service and knowledge users, drivers, and partners in a shared approach to services, a person-centered framework. This requires collaboration, shared decision-making, and shared budgets to deliver services where the person is truly at the center [6,7].

Rehabilitation targets an individual’s needs and priorities in functioning across the continuum of care and throughout the individual’s life span. As countries move toward integrated person-centered care, it is imperative that quality rehabilitation be embedded in service delivery models [8]. The complexity of functioning should be acknowledged in relation to the context in which a person is striving to fulfill their life roles and goals [9,10]. There is a need for interprofessional, person-centered approaches within integrated community-based services to reform the rehabilitation and education of the health workforce [11]. By using technology, people with disabilities can share their functioning in their environment and make an impact by identifying their specific needs, both at an individual and population level [10,12]. Innovative technology, interprofessional teamwork, task shifting, and task sharing are recommended as catalysts for the reform.

Digital technologies are considered an essential solution to health services in countries with limited resources around the world, including those in sub-Saharan Africa [13,14]. Sub-Saharan Africa is home to approximately 1.2 billion people, half of whom are expected to be 25 years or younger by 2050. The region has the world’s largest free trade area, is diverse, and offers human and natural resources that have the potential to yield inclusive growth. Sub-Saharan Africa is composed of 48 low, lower-middle, upper-middle, and high-income countries from Central, Eastern, Southern, and Western Africa, 22 of which are fragile or conflict-affected [15].

In the global context, rehabilitation models are costly and based on professionally delivered services. Most LMICs do not have a sufficiently educated rehabilitation workforce, leading to a situation where services do not meet demand [3]. Digital rehabilitation interventions hold significant potential to address key rehabilitation challenges [16], such as reduction in health care costs [17] and improving treatment adherence, functioning, and quality of life [18]. Strategically leveraging these interventions to address the needs of users directly and equipping different social and health systems with rehabilitation expertise could increase access. This will make present health care systems, especially in low-resource settings, more scalable, inclusive, and resilient in situations where systems become ineffective, overloaded, or do not meet demand [19].

### Goal of This Study

We consider digital rehabilitation as an umbrella term, including different digital technologies that are used by rehabilitation professionals or self-driven service users as part of the rehabilitation process in its different phases (assessment, goal setting, intervention, reassessment, and secondary prevention). These include, but are not limited to, the use of tele- and distance rehabilitation applications and services [20] and the use and design of emerging technologies, such as artificial intelligence–powered conversational agents, wearable devices, emails, video, speech, and text messaging solutions. Digital rehabilitation aims to optimize functioning and reduce disability in individual’s interaction with their environment. Digital rehabilitation has a strong link with the empowerment of individuals and communities (adapted from WHO [1]). The core is connecting and empowering persons and populations to improve functioning and well-being and managing rehabilitation needs.

A multidisciplinary approach considers the person and their entire needs. Rehabilitation is simply not a health issue but includes education, social, livelihood, and empowerment-related efforts [21]. If considered from the professionals’ perspective, it means all professional groups related to rehabilitation, such as physiotherapists, occupational therapists, prosthetist-orthotists, speech therapists, and social workers.

Wider adoption of digital solutions has the potential to improve access to rehabilitation services and avail more research opportunities, especially in LMICs where there is a greater burden and need for rehabilitation services [22,23]. Some of the challenges hindering the adoption of digital rehabilitation identified in sub-Saharan African countries include inadequate and underdeveloped infrastructures for power supply, internet coverage, and transport; the need to train more professionals and enhance patient literacy; the financial implications of transitioning to digital services for health institutions; and the affordability of smart devices for patients [14,24]. However, the extent of research on digital solutions for rehabilitation in the region has not been mapped. There are also a limited number of studies in LMICs about using digital services in rehabilitation. For example, in a review about the use of mobile health applications for stroke, healthy aging, Parkinson disease, and multiple sclerosis, only 5 studies out of more than 130 were from Africa [22].

We conducted a preliminary search of MEDLINE, the Cochrane Database of Systematic Reviews, and JBI Evidence Synthesis and did not identify current or ongoing systematic or scoping reviews on the topic. Unlike Kipruto et al’s [25] ongoing scoping review aiming to map the digital health landscape and the stage of development in sub-Saharan Africa, our study specifically focuses on peer-reviewed digital rehabilitation research not limited to health contexts but includes social, educational, and community settings as well. A recent scoping review by Nizeyimana et al [26] investigated the feasibility and cost-effectiveness of telerehabilitation in a global context. The review focused on both high-income countries and LMICs, considering the accessibility of rehabilitation services and the facilitators and barriers of telerehabilitation.

Therefore, the objectives of this scoping review are to identify the scope and types of evidence of digital rehabilitation solutions and to examine how research about digitally delivered rehabilitation interventions is conducted in sub-Saharan Africa. We expect the review to summarize the available evidence of digital rehabilitation interventions and identify potential research gaps and avenues for development. We chose a scoping review to explore the extent of literature on the topic and as a necessary step in identifying how digital rehabilitation technologies can be integrated into primary health care.

## Methods

### Overview

We will conduct this review according to Arksey and O’Malley’s [27] framework with the 5 mandatory stages and the Joanna Briggs Institute methodology for scoping review guidelines [28]. According to the framework, the stages in a scoping review include (1) identifying the research question, (2) identifying relevant studies, (3) selecting studies, (4) charting the data, and (5) summarizing and reporting the results. A scoping review is used to identify the key concepts relating to the research question, understand the conceptual boundaries of the research topic, and clarify definitions [29].

### Review Question

The main research question guiding this review is: “What is the scope of research related to digital interventions used for rehabilitation in sub-Saharan Africa, and how is the research conducted?”

### Eligibility Criteria

#### Overview

To select relevant studies, we have developed eligibility criteria (Textbox 1). These criteria are structured according to the population, concept, and context categories for scoping reviews suggested by Peters et al [28].

**Eligibility criteria structured according to the population, concept, and context domains. These outline the study population, the concept of digital rehabilitation, and sub-Saharan Africa as the locations of interest.**

**Inclusion criteria**

1. Population: health professionals and patients
  Social, health, and other professionals delivering rehabilitation services
  Persons using or in need of rehabilitation services
2. Concept: use, experiences, and issues of integrating digital services in rehabilitation
  Studies describing the use of digital interventions in rehabilitation
  Studies evaluating the effectiveness of various types of digital rehabilitation interventions
  Studies reporting the problems and barriers to integrating digital services into rehabilitation
3. Context: digital rehabilitation interventions adapted, piloted, and deployed in sub-Saharan Africa
  Studies conducted in health, social, educational, and community settings in sub-Saharan Africa
4. Types of sources
  Peer-reviewed articles, including research protocols
5. Other considerations
  Published in English
  Full article is available on the internet

**Exclusion criteria**

1. Concept
  Studies reporting digitalization of health services other than rehabilitation
2. Types of sources
  Editorials
  Opinion and perspective articles
  Abstracts only
  Literature reviews

#### Population

The review will include studies involving patients of all ages receiving treatment through digital rehabilitation interventions delivered by multidisciplinary rehabilitation professionals.

#### Concept

The concepts of interest in this review are the use and effectiveness of digital rehabilitation interventions and how these interventions are implemented and evaluated in research. For the purposes of the review, we will only include digital rehabilitation interventions, such as telerehabilitation, wearable devices, robotics, virtual and augmented reality, smart device apps, and gamified mobile and web-based interventions.

#### Context

Studies conducted within any rehabilitation setting in sub-Saharan Africa will be eligible for inclusion.

### Evidence Sources

The search strategy will be limited to peer-reviewed literature. Primary research studies of all designs, including both quantitative and qualitative, will be included in this review. Secondary research studies (eg, systematic reviews) will not be included.

### Search Strategy

We planned a search strategy to identify a comprehensive list of research literature specific to digital solutions used for rehabilitation in sub-Saharan Africa. Text in the title and abstracts of relevant articles, and the index terms used to describe the articles, were used to develop a full search strategy for MEDLINE (EBSCO; Multimedia Appendix 1). We will adapt the search strategy with assistance from an information specialist for each included database and information source. We will search databases (MEDLINE [EBSCO], CINAHL, Cochrane Library, and Scopus) for peer-reviewed scientific publications. The reference list of all identified sources will be screened for additional studies.

### Source of Evidence Selection

We will collate and upload identified citations from all databases into Covidence (Veritas Health Innovation) [30] and remove duplicates. Following a pilot test, 2 or more independent reviewers will screen the title and abstract of all retrieved citations for assessment against the inclusion criteria for the review. In the second step, 2 or more independent reviewers will then assess the full-text articles against the inclusion criteria. We will report reasons for the exclusion of sources of evidence in the full text that do not meet the inclusion criteria in the scoping review. We will resolve any disagreements that arise between the reviewers at each stage of the selection process through discussion or with additional reviewers. The results of the search and the study inclusion process will be reported in full in the final scoping review and presented in a PRISMA-ScR (Preferred Reporting Items for Systematic Reviews and Meta-Analyses extension for Scoping Review) flow diagram and checklist by Tricco et al [31].

### Data Extraction

The research team will develop a data extraction tool to record the key information from the studies. Data will be extracted from papers included in the scoping review by 2 or more independent reviewers using the data extraction tool. The data extracted will include specific details about the participants, concept, context, study methods, and key findings relevant to the review question. These include author, publication year, publication type, country of origin, aims, population sample size and characteristics, methodology or methods, intervention type, outcomes and details of these, and other key findings that relate to the review’s question as suggested by the Joanna Briggs Institute [28]. A draft extraction sample is provided in Textbox 2.

The research team will review the data extraction form and pretest it before implementation to ensure it is capturing relevant information. We will make modifications to the form after testing if necessary and state these modifications in the review. The extraction strategy will also be updated according to the categories emerging as the review progresses. We will resolve any disagreements that arise between reviewers through discussion and consensus, or with additional reviewers. The authors of the original papers will be contacted to request missing or additional data when required.

**Data extraction form.**

1. Name of author, date of publication
2. Aims of the study and research question
3. Population
  Characteristics of participants
  Sample size
4. Country of origin (geography of the study)
5. Methodology
  Study design
  Recruitment setting
  Intervention type, comparator, and details of these (eg, duration of the intervention; if applicable).
  Sampling strategy (if applicable)
6. Key findings relating to the research question.
7. Conclusions

### Data Analysis and Presentation

We will summarize the results using descriptive statistics. Descriptive statistics will be used to report the number of studies under each general category and additional conceptual categories, such as the study design, type of intervention, and the challenges and barriers to integrating digital solutions.

A narrative summary will accompany the tabulated and charted results and will describe how the results relate to the review’s objectives and questions. Thematic analysis conducted according to Braun and Clarke’s [32] outline will be used to contextualize the findings and describe the evidence and scope of the digital solutions. The process consists of developing initial coding ideas from the data, manually coding and matching codes to extracts, and sorting the codes into potential themes. Then analyzing the relationships between the themes, reviewing and refining them, and finally tabulating all codes under the final themes [32].

### Ethical Considerations

We will collect all data through searches of web-based scientific databases. No information on individuals will be collected, so approval from a research ethics committee is not required. The study follows the ethical principles of Jamk University of Applied Sciences.

## Results

The protocol was registered and submitted for publication in May 2023. Databases will be searched in September 2023. Based on the preliminary search (Multimedia Appendix 1), we expect to have a reasonable number of references for screening. Screening and data extraction will be done between October and December 2023. The results will be synthesized and reported under the key conceptual categories of this review, and we expect the final scoping review to be ready for submission in early 2024.

## Discussion

### Overview

The review will examine published peer-reviewed research about digital solutions for rehabilitation to establish the extent of their use. This will provide a comprehensive picture of the current knowledge on the use of digital rehabilitation interventions in sub-Saharan Africa and the potential for these interventions to improve patient outcomes. The review extends previous research by Kipruto et al [25] and Nizeyimana et al [26] by emphasizing the “multimodal, patient-centered, and collaborative process” [33] of rehabilitation in a broader health care context, focusing on services to address the health care needs of individuals [1,8,33]. Unique to the present study is the focus on digital solutions for multidisciplinary rehabilitation in low-resource settings, which represents an underexamined topic.

This work is the initial step in a research project aiming to develop evidence-based, person-centered, and context-specific digital rehabilitation services in LMICs in East Africa and Southeast Asia. In the project, we aim to propose a digital-first multidisciplinary approach to address the challenges of access to rehabilitation services in low-resource settings. A digital-first approach will assist in delivering services directly to patients or through the primary health care system instead of solely through rehabilitation professionals, as is the case with the practice in high-income countries.

### Limitations

A potential limitation is the availability of full-text articles. Some relevant articles may not yet be accessible during the search process; thus, limiting the reviewers’ ability to give a summary that reflects all available evidence of digital rehabilitation. Lack of relevant studies because of the sole focus on peer-reviewed research is another limitation. As the digital landscape goes through rapid changes, there may be limited information about studies related to digital rehabilitation interventions in the sub-Saharan context if they, for example, do not progress beyond the trial phase of a project after deployment. Focusing solely on English-only articles also limits the review, as there may be relevant publications in other languages spoken in the sub-Saharan African region, such as French or Portuguese.

We expect the review to give us insights to develop the digital-first rehabilitation model proposed in the project. In addition, we expect it to outline research gaps and the implications for future research, policy, and practice. Other researchers, health professionals, and policy makers will benefit from the results. We will disseminate our findings through different channels, including peer-reviewed publications, presentations at scientific conferences, and web-based blogs.

## Appendix

Multimedia Appendix 1 Search strategy for Medline (EBSCO).

## Abbreviations

LMIC: low- and middle-income country
PRISMA-ScR: Preferred Reporting Items for Systematic Reviews and Meta-Analyses extension for Scoping Review
WHO: World Health Organization

## Data Availability

The data sets generated and analyzed during this study will be included as multimedia appendices in the resulting manuscript.
