# Supplementary material for: Digital Rehabilitation Interventions in Sub-Saharan Africa: Protocol for a Scoping Review
Source: JMIR Res Protoc. 2023 Nov 23;12:e48952. doi: 10.2196/48952 (PMC10704304; doi:10.2196/48952)
Supplement: Multimedia Appendix 1 [file resprot_v12i1e48952_app1.pdf]

Appendices

Appendix I: Search strategy for Medline (EBSCO)

Concept	#	Query	Results May 12th 2023
Rehabilitation and disability	S1	(MH "Disabled Persons") OR (MH "Disabled Children") OR (MH "Developmental Disabilities") OR (MH "Learning Disabilities") OR (MH "Intellectual Disability") OR TI (disabilit* or disabled) OR AB (disabilit* or disabled)	336938
	S2	(MH "Rehabilitation") OR (MH "Psychiatric Rehabilitation") OR (MH "Cardiac Rehabilitation") OR (MH "Rehabilitation Nursing") OR (MH "Rehabilitation Centers") OR (MH "Neurological Rehabilitation") OR (MH "Hospitals, Rehabilitation") OR (MH "Rehabilitation, Vocational") OR (MH "Mouth Rehabilitation") OR (MH "Stroke Rehabilitation") OR (MH "Rehabilitation Research") OR (MH "Physical and Rehabilitation Medicine") OR (MH "Rehabilitation of Speech and Language Disorders") OR (MH "Correction of Hearing Impairment")	64806
	S3	TI rehabilitation OR AB rehabilitation	191561
	S4	(MH "Physical Therapy Modalities") OR (MH "Physical Therapy Specialty") OR (MH "Physical Therapy Department, Hospital") OR (MH "Physical Therapist Assistants") OR (MH "Exercise Therapy") OR (MH "Exercise") OR (MH "Preoperative Exercise") OR TI ("physical therap*" or physiotherap* or "exercise therap*" or "exercise intervention" or "therapeutic exercise") OR AB ("physical therap*" or physiotherap* or "exercise therap*" or "exercise intervention" or "therapeutic exercise")	258713
	S5	(MH "Occupational Therapy") OR (MH "Occupational Therapy Department, Hospital") OR (MH "Occupational Therapists") OR TI (occupation* N4 (therap* or rehabil*)) OR AB ((occupation* N4 (therap* or rehabil*))	25037
	S6	(MH "Psychotherapy+") OR TI psychotherap* OR AB psychotherap*	233069
	S7	(MH "Recreation Therapy") OR (MH "Dance Therapy") OR (MH "Music Therapy") OR (MH "Art Therapy") OR (MH "Speech Therapy") OR TI ("recreation* therap*" or "dance therap*" or "music therap*" or "art therap*" or reablement or "speech therap*") OR AB ("recreation* therap*" or "dance therap*" or "music therap*" or "art therap*" or reablement or "speech therap*")	29636
	S8	S1 OR S2 OR S3 OR S4 OR S5 OR S6 OR S7	976639
Digital rehabilitation	S9	(MH "Telemedicine") OR (MH "Telehealth") OR (MH "Internet-Based Intervention") OR TI (ehealth* or e-health* or mhealth*	57620

		or m-health or "mobile health" or telehealth* or telemedicine) OR AB (ehealth* or e-health* or mhealth* or m-health or "mobile health" or telehealth* or telemedicine)	
	S10	(MH "Virtual Reality") OR (MH "Exergaming") OR (MH "Video Games") OR (MH "Robotics") OR (MH "Virtual Exposure Therapy") OR (MH "Internet+") OR (MH "Digital Technology") OR (MH "Cell Phone") OR (MH "Telecommunications") OR (MH "Smartphone") OR (MH "Mobile Applications") OR TI ("virtual reality" or "virtual environment" or "augmented reality" or technolog* or exergame or gamifi* or "video game" or robot* or software or computer or internet) OR AB ("virtual reality" or "virtual environment" or "augmented reality" or exergame or gamifi* or "video game" or robot* or computer or internet)	598786
	S11	S9 OR S10	638815
	S12	S8 AND S11	39504
	S13	TI ((distan* or remote* or tele* or online* or digital* or mobile or application) N3 (rehabilitation or "physical therap*" or physiotherap* or "exercise therap*" or "exercise intervention" or "therapeutic exercise" or (occupation* N4 (therap* or rehabil*)) or "recreation* therap*" or "dance therap*" or "music therap*" or "art therap*" or reablement or "speech therap*" or psychotherap*)) OR AB ((distan* or remote* or tele* or online* or digital* or mobile or application) N5 (rehabilitation or "physical therap*" or physiotherap* or "exercise therap*" or "exercise intervention" or "therapeutic exercise" or (occupation* N4 (therap* or rehabil*)) or "recreation* therap*" or "dance therap*" or "music therap*" or "art therap*" or reablement or "speech therap*" or psychotherap*))	7037
	S14	(MH "Telerehabilitation") or TI telerehabilitation or AB telerehabilitation	1867
	S15	S12 OR S13 OR S14	44883
Sub Saharan Africa	S16	(MH "Africa South of the Sahara") OR TI ("sub Saharan Africa" OR “sub-Saharan Africa” OR “sub-Sahara” OR sub-Sahara OR SSA OR “Africa South of the Sahara” OR Angola OR Benin OR Botswana OR “Burkina Faso” OR Burundi OR Cameroon OR “Cape Verde” OR Cameroon OR “Central African Republic” OR Chad OR Comoros OR Congo OR “Côte d'Ivoire” OR Djibouti OR “Equatorial Guinea” OR Eritrea OR Eswatini OR Ethiopia OR Gabon OR “The Gambia” OR Ghana OR Guinea OR “Guinea-Bissau” OR Kenya OR Lesotho OR Liberia OR Madagascar OR Malawi OR Mali OR Mauritania OR Mauritius OR Mozambique OR Namibia OR Niger OR Nigeria OR Rwanda OR “Sao tome and Principe” OR Senegal OR Seychelles OR “Sierra Leone” OR Somalia OR “South Africa” OR “South Sudan” OR Sudan OR Tanzania OR Togo OR Uganda OR Zambia OR Zimbabwe) OR AB ("sub Saharan Africa" OR “sub-Saharan Africa” OR “sub-Sahara”	400695

		OR sub-Sahara OR SSA OR “Africa South of the Sahara” OR Angola OR Benin OR Botswana OR “Burkina Faso” OR Burundi OR Cameroon OR “Cape Verde” OR Cameroon OR “Central African Republic” OR Chad OR Comoros OR Congo OR “Côte d'Ivoire” OR Djibouti OR “Equatorial Guinea” OR Eritrea OR Eswatini OR Ethiopia OR Gabon OR “The Gambia” OR Ghana OR Guinea OR “Guinea-Bissau” OR Kenya OR Lesotho OR Liberia OR Madagascar OR Malawi OR Mali OR Mauritania OR Mauritius OR Mozambique OR Namibia OR Niger OR Nigeria OR Rwanda OR “Sao tome and Principe” OR Senegal OR Seychelles OR “Sierra Leone” OR Somalia OR “South Africa” OR “South Sudan” OR Sudan OR Tanzania OR Togo OR Uganda OR Zambia OR Zimbabwe)	
	S18	S14 AND S15	232
